# Soil nematode metacommunities in different land covers: Assessment at the local and regional scales

**DOI:** 10.1002/ece3.11468

**Published:** 2024-05-23

**Authors:** Ximei Niu, Ping Wang, Zhijing Xie, Meixiang Gao, Siru Qian, Ruslan Saifutdinov, Nonillon M. Aspe, Donghui Wu, Pingting Guan

**Affiliations:** ^1^ State Environmental Protection Key Laboratory of Wetland Ecology and Vegetation Restoration, School of Environment Northeast Normal University Changchun China; ^2^ Key Laboratory of Vegetation Ecology, Ministry of Education Northeast Normal University Changchun China; ^3^ Department of Geography and Spatial Information Techniques Ningbo University Ningbo China; ^4^ Laboratory for Soil Ecological Functions A.N. Severtsov Institute of Ecology and Evolution, Russian Academy of Sciences Moscow Russia; ^5^ College of Marine and Allied Sciences Mindanao State University at Naawan Naawan Philippines; ^6^ Key Laboratory of Wetland Ecology and Environment, Northeast Institute of Geography and Agroecology Chinese Academy of Sciences Changchun China

**Keywords:** biological interactions, dispersal limitation, environmental heterogeneity, metacommunity structure, nematode community assemblage, spatial scales

## Abstract

The metacommunity theory enhances our understanding of how ecological processes regulate community structure. Yet, unraveling the complexities of soil nematode metacommunity structures across various spatial scales and determining the factors influencing these patterns remains challenging. Therefore, we conducted an investigation on soil nematode metacommunities spanning from north to south in the Northeastern China. Our aim was to test whether nematode metacommunities were structured by different drivers under three land covers (i.e., farmland, grassland and woodland) at the local and regional scales. The results revealed that the Clementsian, Gleasonian and their quasi‐structures of soil nematodes collectively accounted for 93% of the variation across the three land covers at the local and regional scales. These structures suggest that the soil nematode metacommunities in the Northeast China responded to fluctuations in environmental gradients. At the local scale, metacommunities were primarily shaped by biological interactions. At the regional scale, environmental heterogeneity, dispersal limitation and biological interactions all contributed to nematode metacommunities. Meanwhile, biological interactions under three land covers were represented within different trophic groups, with plant parasites predominant in farmlands and bacterivores in grasslands and woodlands. In conclusion, the metacommunity structures of soil nematodes remain stable at different spatial scales and land covers. Biological interactions are widespread among nematodes regardless of changes in spatial scales and land covers. This study reveals the importance of nematode sensitivity to the environment and biological interactions in shaping the nematode metacommunities, potentially enhancing our understanding of the spatial patterns of nematode metacommunities.

## Graphical abstract

This study investigated the metacommunity structures of soil nematodes and the mechanisms of their construction under different land covers with the local and regional scales. The results suggested that the metacommunity structures of soil nematodes remained stable at different spatial scales and land covers. In addition to environmental filtering and dispersal limitation, biological interactions were widespread among nematodes regardless of changes in spatial scales and land covers.

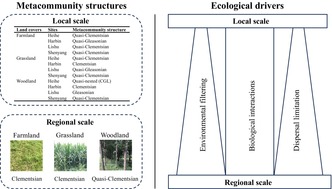

## 1 INTRODUCTION

Understanding community assembly is one of the primary goals in studying community ecology (Pachepsky et al., 2001). However, due to the spatial complexity of habitats, including variations in structures and patterns, there is no direct and definite way to understand community ecology (Heino et al., 2015). The metacommunity theory has increasingly been used to identify the drivers of community assembly for different organisms (Murray‐Stoker & Murray‐Stoker, 2020; Wisnoski & Lennon, 2021; Zheng & Yin, 2023).

Metacommunity theory, as a fundamental concept in community ecology, links the local and regional scales and considers the interactions among species (Thompson et al., 2020). It refers to a group of local communities connected through the dispersal of multiple species, all of which have the potential to interact with each other (Leibold et al., 2004). In the development of metacommunity theory, six main structural types have been identified: (1) the Clementsian structure represents a gradient wherein species collectively respond to ecological gradients, leading to discrete communities (Clements, 1916); (2) the Gleasonian structure arises from specific responses of species to environmental changes (Gleason, 1926); (3) nestedness formation is linked to variations in species dispersal abilities, habitat specialization and abiotic tolerances (Patterson & Atmar, 1986); (4) strong interspecific competition may result in a trade‐off in competitive abilities, leading to a more evenly spaced distribution along environmental gradients (Tilman, 1982); (5) however, intense competition can yield checkerboard structures among paired species with mutually exclusive ranges (Diamond, 1975); and (6) random structure is utilized to describe species distributions lacking gradients or pattern changes (Simberloff, 1983). Each structure represents a prediction regarding the relationship between ecological mechanisms and species distribution (Leibold & Mikkelson, 2002). The analysis of elements of metacommunity structures has been confirmed to be an effective way to explain the above structures (Gao et al., 2016; Li et al., 2021). Identifying the drivers of community assembly is equally important aside from determining the metacommunity structures. In community ecology, faunal composition can be explained by the environmental heterogeneity, dispersal limitation and biological interactions (Leibold et al., 2004; Thompson & Townsend, 2006). For example, it has been shown in Puzin et al. (2019) that the dispersal of soil macroorganisms is influenced by their individual density and habitat availability at different spatial scales. Markfeld et al. (2022) showed that the impact of patch connectivity on soil arthropod diversity in the agricultural ecosystem is spatially dependent and the stability of metacommunities in different taxonomic groups varies with the spatial scales (Wisnoski et al., 2023). However, it is still unclear how the metacommunities of soil organisms vary with different spatial scales.

Soil nematodes are among the most abundant metazoans in soil communities, with a wide distribution, accounting for four‐fifths of the terrestrial fauna (van den Hoogen et al., 2019). They are categorized into different trophic groups, and each group plays an important role in belowground ecosystems (Neher, 2010; Yeates et al., 1993). These microscopic invertebrates are sensitive to environmental changes and serve as indicator organisms that reveal soil functions (Martin et al., 2022). It had been documented that nematode metacommunities exhibit strong spatial dependence (Dümmer et al., 2016). In the freshwater ecosystems, environmental heterogeneity is a key factor of nematode metacommunity as shown in a mesocosm experiment by Gansfort et al. (2021). The dispersal process is another important driver for the metacommunity of passive dispersal nematodes (Ptatscheck et al., 2020). There is a growing evidence that environmental heterogeneity and dispersal limitation differentially drive the metacommunity structures of nematodes in aquatic ecosystems (Gansfort & Traunspurger, 2019; Hauquier et al., 2018). However, these studies have been focused on the aquatic ecosystems (Alejandro Cisterna‐Celiz et al., 2019; Gansfort et al., 2020). Relatively few studies have investigated the metacommunity assembly of nematodes in the terrestrial ecosystems. For example, Scharroba et al. (2016) showed that nematode metacommunities across different depths are influenced by the availability and quality of resources. However, the relative importance of environmental heterogeneity and dispersal limitation remain unclear for the metacommunity structures of soil nematodes in terrestrial ecosystems. The biological interactions of nematodes play another important role in the coexistence of soil fauna (Caruso et al., 2019). Yet only few studies have focused on evaluating the effect of biological interactions on soil nematode metacommunity assembly, leading to their underestimation. Therefore, aside from environmental heterogeneity and dispersal limitation, investigating the biological interactions of soil nematodes is also important. Soil nematodes living in the water of soil pores are sensitive to environmental changes (Chen et al., 2021), and their composition is significantly influenced by land covers at different scales (Li, Chen, et al., 2022; Li, Liu, et al., 2022; Luo et al., 2022). The structures of nematode metacommunities and their assembly mechanisms could change in different land covers (Brustolin et al., 2022). Therefore, though challenging, exploring the metacommunity structures of soil nematodes at different spatial scales with multiple land covers would shed light to these interactions.

Understanding the metacommunity structures of soil nematodes is essential for maintaining coexistence and comprehending the spatial pattern of soil nematodes (Daly et al., 2021; Spedicato et al., 2023). In this study, the main objectives were to discover the metacommunity structures of soil nematodes and their construction mechanism among three typical land covers (i.e., farmland, grassland and woodland) at the local and regional scales. We hypothesized that: (1) considering that the farmlands are heavily disturbed artificial management system, the environment in the farmlands are often homogeneous and might have lower environmental heterogeneity. The low environmental heterogeneity could result in a less pronounced distribution structure of genera along environmental gradients; the soil nematode metacommunities in the farmlands would show random structures. Since the grasslands and woodlands are natural ecosystems with less disturbance and with higher environmental heterogeneity, the soil nematode metacommunities in these land covers are expected to show non‐random structures; (2) Moreover, the local‐scale effects tend to be linked to interactions between biotic and local abiotic factors (Chase et al., 2020; Cosentino et al., 2023). We hypothesized that the farmlands, exhibiting lower environmental heterogeneity, might primarily influence soil nematode metacommunities through biological interactions at the local scale. In contrast, the grasslands and woodlands, characterized by higher environmental heterogeneity, might be mainly affected by both environmental heterogeneity and biological interactions. Additionally, at the regional scale, dispersal limitation might be a major factor due to the dispersal capacity of nematodes under all three land covers.

## 2 MATERIALS AND METHODS

### 2.1 Study sites

The study was conducted in four long‐term research stations located in the Northeast China, which include the following: Heihe Branch of Heilongjiang Academy of Agricultural Sciences (hereafter referred to as “Heihe”), Soil Fertilizer and Environmental Resources Research Institute of Heilongjiang Academy of Agricultural Sciences (hereafter referred to as “Harbin”), Lishu Experimental Station of China Agricultural University (hereafter referred to as “Lishu”) and Experimental Station of Shenyang Agricultural University (hereafter referred to as “Shenyang”). These sites have a temperate continental monsoon climate. The mean annual temperature (MAT) is 2–11°C, and the mean annual precipitation (MAP) is 500–700 mm, of which 70% is concentrated in July–August (Xie et al., 2019). Brief description of the study sites, including the mean annual temperature, the mean annual precipitation and soil types, is provided in Table 1.

**TABLE 1:** Brief description of the study sites, including geographic coordinates, mean annual temperature (MAT), mean annual precipitation (MAP), and soil type.

Site	Geographic coordinate	MAT (°C)	MAP (mm)	Soil type
Heihe	50°15′ N,127°27′ E	2.0	510	Dark brown soil
Harbin	45°40′ N,126°35′ E	5.5	500	Black soil
Lishu	43°21′ N,124°37′ E	5.8	576	Black soil
Shenyang	40°48′ N,123°33′ E	7.9	700	Brown soil

### 2.2 Experimental design and sampling

Soil samples were collected in the growing season of 2019 and 2021. In each site, three land covers, which include farmland (monoculture planted with corn), grassland and woodland, were selected. The research design incorporated two spatial scales: the local scale and the regional scale. At each land cover (farmland, grassland and woodland), each site (Heihe, Harbin, Lishu and Shenyang) was treated as the local scale. Combining all four sites together was considered as the regional scale. Three replicate plots, each measuring 20 × 20 m, were selected for each land cover, with 5 m between each plot. Five 1 × 1 m subplots were set up in each plot. Soil samples were collected from each subplot with a 2.5 cm diameter soil corer to a depth of 10 cm. Samples from five subplots within one plot were combined to create one true replicate. After collecting, samples were stored at 4°C for less than one week before subsequent extraction of soil nematodes and measurement of environmental variables. Geographic information, including the longitude and latitude of each plot, was recorded for further analysis (Table 1). In total, the experiment consisted of 4 sampling sites × 3 land covers × 3 replications × 2‐year sampling = 72 samples.

### 2.3 Environmental and spatial variables

Soil samples were dried to a constant weight at 105°C to determine soil moisture (SM). Soil temperature (ST) was measured by a soil moisture and temperature sensor (Shunkeda TR‐8D, China). Soil pH was measured in a 1:2.5 soil–water solution with a pH meter (Thermo Fisher Scientific Inc., USA). Soil total carbon (STC) and soil total nitrogen (STN) were determined by the combustion method using an automatic elemental analyzer (Elementar Analyzer System Vario MACRO CUBE, German). Soil total phosphorus (STP) was determined by the ablation method using an inductively coupled plasma atomic emission spectrometer (Thermo ICAP 6000 SERIES, USA). Spatial variables include the longitude and the latitude.

### 2.4 Soil nematode extraction and identification

Nematodes were extracted from 50 g of fresh soil using the Oostenbrink cotton‐wool filter method (Oostenbrink, 1960) with modifications described by Townshend (1963). The nematode samples were immersed in a 4% (volume per volume) formaldehyde solution. Soil nematodes were counted and identified under an inverted microscope (MODEL ECLIPSE Ts2, Japan). One hundred nematodes were randomly selected from each sample and identified to the genus level, with fewer than 100 being fully identified. Nematodes were identified using “De nematoden van Nederland” (Bongers, 1994) and the following information given on website http://nemaplex.ucdavis.edu/Uppermnus/topmnu.htm. After identification, all nematodes were classified into the following trophic groups according to their feeding preferences: plant parasites, bacterivores, fungivores and omnivores–predators (Neher, 2010; Yeates et al., 1993). Due to the challenges associated with nematode identification and the broad applicability of nematode genera in ecology studies, this study employed genus‐level analysis of nematode metacommunity.

### 2.5 Statistical analysis

All statistical analyses were performed in R software version 4.0.3 (R Core Team, 2021).

#### 2.5.1 Elements of metacommunity structure

The analysis of elements of metacommunity structure (EMS) was employed to examine the metacommunity structures of soil nematodes. The calculations involved initial conversion of site‐genus‐based abundance matrix into a presence–absence matrix. These matrices were subsequently ranked using the reciprocal averaging (RA) method, arranging the rows and columns to position genera (columns) with the most similar distributions close together, and sites (rows) with the most similar lists of genera adjacent to each other. Based on three indicators (coherence, turnover and boundary clumping), EMS was employed to identify metacommunity structures of soil nematodes (e.g., Clementsian, Gleasonian, nestedness, checkerboard, evenly spaced, random and quasi‐structural patterns; Leibold & Mikkelson, 2002; Presley et al., 2010).

1. Coherence was evaluated by comparing the absence counts between the coordinate matrix and the simulation matrix. The *Z*‐test was utilized to compare the absence counts within a specific embedding. Non‐significant coherence (where the number of embedded absences differed from that expected by chance) suggested a random distribution of the metacommunity. A significantly negative coherence (where the number of embedded absences was higher than expected by chance) indicated a checkerboard distribution pattern. Conversely, a significantly positive coherence indicated fewer absences embedded within the genus range than expected. This implied that the metacommunity was structured along an environmental gradient, necessitating additional analysis and confirmation through turnover and boundary clumping.
2. Turnover was defined as the number of times with which one genus replaced another between two sampling points along a ranking axis. If turnover was non‐significant, then the metacommunity exhibited a quasi‐structure. The significant negative turnover (when the measured turnover was significantly smaller than the expected value) indicated a nested distribution. The significant positive turnover (when the measured turnover was significantly greater than the expected value) referred to a specific distribution and needed to be judged with boundary‐clumping indicators for further analysis.
3. Boundary clumping was quantified using Morisita's index (*I*) and a Chi‐squared test, comparing observed and expected distributions of genera boundaries. The results were interpreted as follows: (i) *I*‐values close to 1 indicated a Gleasonian structure; (ii) *I*‐values significantly greater than 1 suggested a Clementsian structure; and (iii) *I*‐values significantly lower than 1 indicated evenly spaced distribution. All EMS analyses were conducted using the “Metacom” and “vegan” packages (Dallas, 2014).

#### 2.5.2 Mantel test

Mantel test was used to assess the importance of environmental heterogeneity (environmental variables) and dispersal limitation (spatial variables) on soil nematode metacommunities. The dissimilarity of soil nematode metacommunity was assessed using the Bray–Curtis distance, whereas both environmental and spatial variables were measured using the Euclidean distance. The Mantel test was calculated by the “geosphere.”

#### 2.5.3 Random forest

A byproduct of the ordination procedure were the site scores, which were used to rank sites and genera in the presence–absence matrix. The scores represented structural gradients that can be correlated with different variables (Presley & Willig, 2010). Random forest (Liaw & Wiener, 2001) was employed to validate significant correlations between site scores and environmental and spatial variables calculated by the “randomForest” package.

#### 2.5.4 Analysis of co‐occurrence patterns

Co‐occurrence patterns of soil nematode metacommunity were analyzed with the *C*‐score method (Gotelli, 2000; Gotelli & Ulrich, 2012). This involved a null model simulation to determine whether the soil nematode metacommunity displayed random or non‐random coexistence pattern. The *C*‐score quantified the average number of “checkerboard units” among all possible pairs of genera (Stone & Roberts, 1990). These checkerboard patterns in the soil nematode metacommunity reflected whether competitive interactions among genera occur. The standardized effect size (SES) estimated the number of standard deviations between the observed metrics and the simulated metrics (Gotelli & McCabe, 2002).

(1) $$C‐\text{score}=\frac{\sum \left(R_{i}-S\right)\left(R_{j}-S\right)}{\left(\frac{R\left(R-1\right)}{2}\right)}$$

(2) $$\mathrm{SES}=\left(C‐{\text{score}}_{\mathrm{obs}}-C‐{\text{score}}_{\mathrm{sim}}\right)/\left(C‐{\text{score}}_{\mathrm{sim}}\right)$$

*S* is the number of sites where the two genera occur, *R* is the total number of rows of sites containing the two genera, *R*_*i*_ and *R*_*j*_ are the total number of matrix rows of genera *i* and *j*. *C*‐score_obs_ represents the *C*‐score observed value, and *C*‐score_sim_ represents the *C*‐score simulated value. Assuming that SES follows normal distribution, it is expected that 95% of the SES confidence intervals are distributed between −2.0 and 2.0. An SES larger than 2.0 indicates non‐random segregation. This implies that the metacommunity is considered to be a competitive community, suggesting that biological interactions play an important regulatory role in community co‐occurrence. An SES lower than −2.0 indicates non‐random aggregation. We analyzed the co‐occurrence patterns for all genera within the nematode metacommunity. As competition between genera for dietary resources might play an important role in community distribution, we further analyzed the co‐occurrence patterns of different trophic groups (Kamilar & Ledogar, 2011; Stamou & Papatheodorou, 2023). These indices were calculated from 10,000 null randomization matrices. The *C*‐score and SES were performed using the “Ecosim” package.

## 3 RESULTS

### 3.1 Metacommunity structures at the local and regional scales among land covers

At the local scale, the metacommunity structures of soil nematodes showed significant coherence (Abs was significantly lower than the mean). Further analyses of turnover and boundary clumping revealed that the soil nematode metacommunities in farmlands of Heihe, Lishu and Shenyang exhibited Quasi‐Clementsian structure, while a Quasi‐Gleasonian structure was observed in Harbin. For grasslands, the soil nematode metacommunities in Heihe and Shenyang exhibited Quasi‐Clementsian structure. The metacommunity structure was Clementsian in Harbin, while the Quasi‐Gleasonian structure was observed in grasslands of Lishu. Nematode metacommunities of woodlands corresponded to Quasi‐nested (CGL) structure, Clementsian, Gleasonian and Quasi‐Clementsian in Heihe, Harbin, Lishu and Shenyang, respectively. In general, the Clementsian, Gleasonian and their Quasi‐structures were obtained more frequently at the local scale (Table 2). At the regional scale, according to the results of EMS analysis, the soil nematode metacommunities showed the Clementsian structure in the farmlands and grasslands, while the soil nematode metacommunity in the woodlands was Quasi‐Clementsian (Table 2).

**TABLE 2:** Results of the EMS analysis of soil nematodes under three land covers at two different spatial scales.

Metacommunity	Coherence					Turnover					Boundary clumping		Metacommunity structure
	Abs	*p*	Mean	SD	*Z*	Rep	*p*	Mean	SD	*Z*	*I*	*p*	
Local scale													
Farmland													
Heihe	40	**.00**	102.21	4.94	−12.58	639	.08	507.69	75.78	1.73	1.28	**.01**	Quasi‐Clementsian
Harbin	33	**.00**	139.30	5.13	−20.37	1095	.16	949.87	102.87	1.41	1.11	.07	Quasi‐Gleasonian
Lishu	32	**.00**	112.60	5.24	−15.37	764	.08	627.43	79.09	1.73	1.28	**.01**	Quasi‐Clementsian
Shenyang	49	**.00**	134.78	4.93	−17.39	819	.05	682.85	70.56	1.93	1.24	**.01**	Quasi‐Clementsian
Grassland													
Heihe	57	**.00**	185.54	6.99	−18.38	1771	.44	1659.00	145.50	0.77	1.19	**.00**	Quasi‐Clementsian
Harbin	37	**.00**	125.01	4.54	−19.39	1257	**.02**	966.69	127.17	2.28	1.21	**.01**	Clementsian
Lishu	55	**.00**	179.35	6.54	−19.01	1651	.27	1496.86	141.19	1.09	1.01	.28	Quasi‐Gleasonian
Shenyang	65	**.00**	278.34	8.24	−25.89	3184	.69	3115.31	173.00	0.40	1.50	**.00**	Quasi‐Clementsian
Woodland													
Heihe	44	**.00**	162.95	8.87	−13.41	1463	.19	1652.90	143.33	−1.32	1.15	**.01**	Quasi‐nested (CGL)
Harbin	38	**.00**	159.54	4.21	−28.85	1602	**.00**	1241.29	125.16	2.88	1.15	**.02**	Clementsian
Lishu	43	**.00**	190.65	4.96	−29.77	1800	**.00**	1463.85	114.83	2.93	1.07	.10	Gleasonian
Shenyang	70	**.00**	169.00	5.64	−17.55	1247	.39	1148.96	115.19	0.85	1.29	**.00**	Quasi‐Clementsian
Regional Scale													
Farmland	666	**.00**	1162.01	17.13	−28.96	19,264	**.00**	13631.22	1772.41	3.18	1.45	**.00**	Clementsian
Grassland	976	**.00**	1784.21	19.08	−42.35	40,576	**.00**	28797.35	3113.70	3.78	1.71	**.00**	Clementsian
Woodland	945	**.00**	1545.22	19.97	−30.06	30,248	.29	26927.46	3117.31	1.07	1.77	**.00**	Quasi‐Clementsian

*Note*: Significant reported in bold.

Abbreviations: Abs, the number of embedded absences; CGL, clumped genus loss; *I*, Morisita index; Mean, mean value for the null model distribution; *p*, the probability from a *Z*‐test to assess the significance of the observed index to the null matrices; Rep, the number of replacements; SD, standard deviation value for the null model; *Z*, presented for inter‐annual comparison (number of standard deviations from the mean).

### 3.2 Drivers of metacommunity at the local and regional scales among land covers

At the local scale, for farmlands, there was a significant correlation between the metacommunity dissimilarity of soil nematodes and the environmental distance in Harbin (*r* = .71, *p* < .05) and Lishu (*r* = .73, *p* < .05; Table 3). The metacommunity dissimilarity and the spatial distance had a significant correlation in Heihe (*r* = .78, *p* < .001). In case of grasslands, both environmental distance (*r* = .62, *p* < .05) and spatial distance (*r* = .79, *p* < .05) showed significant correlations with the metacommunity dissimilarity of soil nematodes in Harbin. Furthermore, the dissimilarity of metacommunities was only significantly correlated with spatial distance in grasslands of Lishu (*r* = .88, *p* < .05) and Shenyang (*r* = .69, *p* < .05). In woodlands, across all studied sites, only Lishu demonstrated a significant correlation between the dissimilarity of metacommunities and environmental distance (*r* = .56, *p* < .05), as well as spatial distance (*r* = .51, *p* < .05), respectively. At the regional scale, the results of the Mantel test showed that the metacommunity dissimilarity of soil nematodes was significantly correlated with both environmental and spatial distance under three land covers (.14 ≤ *r* ≥ .27, *p* < .05; Table 3).

**TABLE 3:** Relationship between soil nematode metacommunity dissimilarity and environmental distance and spatial distance based on the Mantel test.

Metacommunity	Env		Spa	
	*r*	*p*	*r*	*p*
Local scale				
Farmland				
Heihe	.38	.10	.78	**.00**
Harbin	.71	**.02**	.51	.06
Lishu	.73	**.01**	.64	.08
Shenyang	−.15	.74	−.11	.65
Grassland				
Heihe	.34	.12	.46	.11
Harbin	.62	**.03**	.79	**.01**
Lishu	.67	.08	.88	**.01**
Shenyang	.45	.05	.69	**.01**
Woodland				
Heihe	.11	.36	.44	.09
Harbin	.00	.50	.61	.07
Lishu	.56	**.04**	.51	**.02**
Shenyang	.06	.40	−.28	.78
Regional scale				
Farmland	.20	**.01**	.21	**.01**
Grassland	.27	**.00**	.14	**.04**
Woodland	.26	**.02**	.25	**.00**

*Note*: The Spearman correlation was used for this analysis; significant correlations are reported in bold.

Abbreviations: Env, environmental distance; Spa, spatial distance.

**FIGURE 1:**
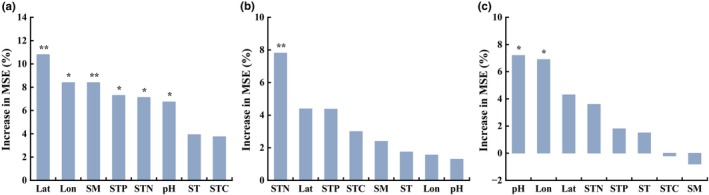
Results of Random Forest analysis with site scores of the nematodes presence–absence matrix in relation to environmental and spatial variables across three land covers. (a) farmlands; (b) grasslands; (c) woodlands. The bars are ranked from greatest to least MSE. Lat, latitude; Lon, longitude; MSE, mean squared error; SM, soil moisture; ST, soil temperature; STC, soil total carbon; STN, soil total nitrogen; STP, soil total phosphorus. ^ns^ *p* > .05; *p < .05; **p < .01; ***p < .001.

The results of random forest at the regional scale showed that the soil nematode metacommunity in the farmlands was significantly influenced by latitude, longitude, SM, STP, STN and pH (*p* < .05; Figure 1a). The nematode metacommunity in the grasslands was significantly affected by STN (*p* < .05; Figure 1b). Soil pH and longitude significantly influenced nematode metacommunity in the woodlands (*p* < .05; Figure 1c).

**FIGURE 2:**
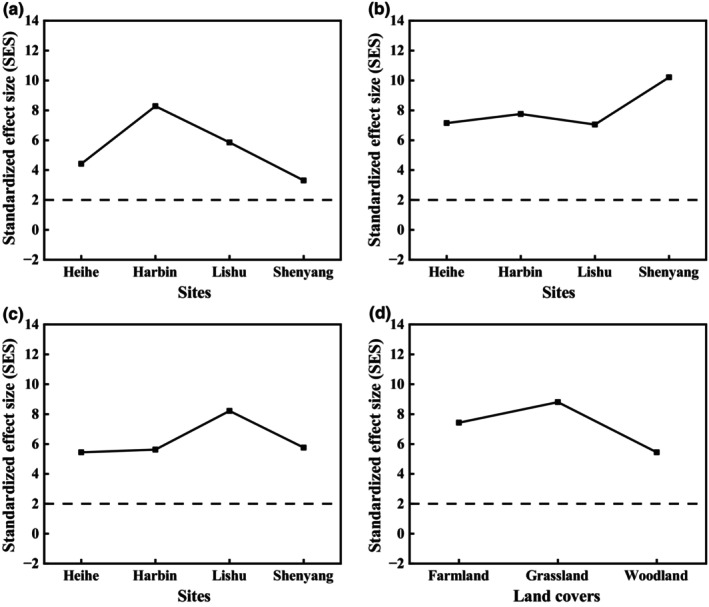
Co‐occurrence patterns of soil nematode metacommunity: results of null model analysis. The standardized effect size (SES) larger than 2.0 indicates non‐random segregation, whereas the SES lower than −2.0 indicates non‐random aggregation. (a) local farmlands; (b) local grasslands; (c) local woodlands; (d) various land cover types at the regional scale.

**FIGURE 3:**
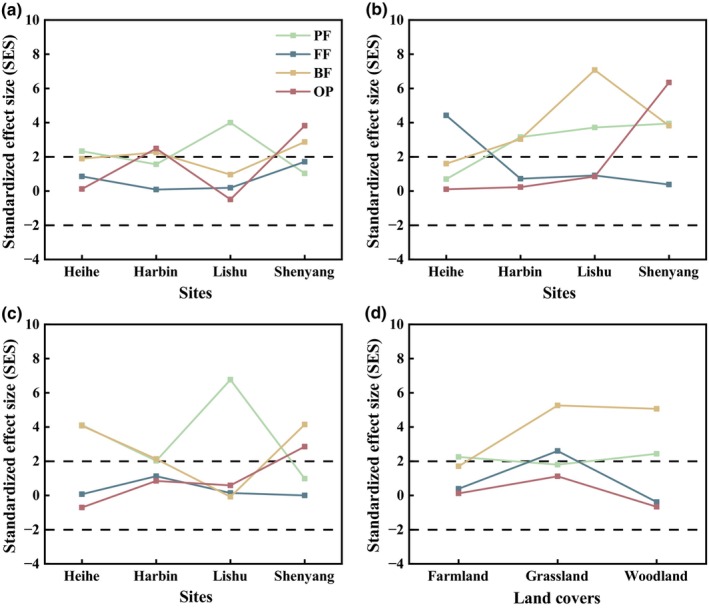
Co‐occurrence patterns of different trophic groups of soil nematode metacommunity: results of null model analysis. (a) local farmlands; (b) local grasslands; (c) local woodlands; (d) various land cover types at the regional scale. BF, bacterivores; FF, fungivores; OP, omnivores–predators; PF, plant parasites.

At the local and the regional scales, the analysis of co‐occurrence patterns based on the null model showed that all SESs were greater than 2 (Figure 2), indicating non‐random segregation among the soil nematode metacommunities. At the local scale, for farmlands, plant parasites in Heihe and Lishu, along with omnivores–predators and bacterivores in Harbin and Shenyang, exhibited high SES values of over 2 (Figure 3a). For grasslands, the SESs of fungivores in Heihe, bacterivores and plant parasites in Harbin and Lishu, omnivores–predators and plant parasites and bacterivores in Shenyang were greater than 2 (Figure 3b). As for woodlands, plant parasites and bacterivores in Heihe and Harbin, plant parasites in Lishu, bacterivores and omnivores–predators in Shenyang showed SES values over 2 (Figure 3c). At the regional scale, only plant parasites had the SES values greater than 2 in the farmlands. The SESs of bacterivores and fungivores in the grasslands and bacterivores and plant parasites in the woodlands were greater than 2. In the grasslands and woodlands, the SES of bacterivores had higher values than the other trophic groups (Figure 3d).

## 4 DISCUSSION

### 4.1 Metacommunity structures remain stable at different spatial scales and land covers

The metacommunity structures of soil nematodes demonstrated stability across different spatial scales and land cover types. The soil nematode metacommunities at the local scale showed a mixture of Clementsian, Gleasonian and their Quasi‐structures, irrespective of land cover types. Both the Clementsian and Gleasonian structures were significantly influenced by the environmental gradients. The Clementsian structure suggests that nematode communities replace each other as a group (Leibold & Mikkelson, 2002; Presley et al., 2010) and also implies that the composition of nematode communities varies continuously along environmental gradients at the genus level. The Clementsian structure appeared when nematode communities responded similarly to the environmental gradient. The Gleasonian structure indicates that the distribution of the nematode community is random (Lopez‐Gonzalez et al., 2012). This could be attributed to the unique changes exhibited by nematodes in response to abiotic factors, with coexistence arising from accidental similarity in demand and tolerance (Presley et al., 2010). This suggests that the nematode metacommunities were influenced by environmental heterogeneity, with nematode genera exhibiting different responses to the environmental gradient at the local scale. Additionally, most of them belonged to quasi‐structures. The presence of quasi‐structures suggests that the niche width of the genus exceeded the range of significant turnover at the local scale (Diniz et al., 2021). The Clementsian, Gleasonian and their Quasi‐structures were observed more frequently at the local scale in this study, indicating that environmental heterogeneity at the local scale played a significant role in shaping soil nematode metacommunities (He et al., 2020).

At the regional scale, the metacommunity structures of soil nematodes were similar to those observed at the local scale, with all three land covers exhibiting Clementsian, Clementsian and Quasi‐Clementsian structures. This suggests that the soil nematode metacommunity was primarily influenced by soil environmental variables at the regional scale. Under each land cover, high environmental heterogeneity was created by spanning the geographic range of sites (Henriques‐Silva et al., 2013). It has been shown in the works of Guo et al. (2018) and Li et al. (2021) that the metacommunities of ground‐dwelling macro‐arthropods was Clementsian at the regional scale. Our study showed similar findings for nematodes. This may happen due to the sensitivity of nematodes to the environment. Given their tendency to markedly change with environmental gradients, nematode metacommunities exhibited Clementsian structures (Urzelai et al., 2000). Furthermore, although it was commonly assumed that agricultural management intensity led to significant changes in soil organism community (Gong et al., 2023; Gupta et al., 2022), contrary to our first hypothesis, the soil nematode metacommunity in farmlands still exhibited significant coherence and non‐random structures. The soil nematode metacommunity was not significantly influenced by land cover at each spatial scale. This indicates that, regardless of land cover types, soil nematode metacommunity was more dependent on the environmental heterogeneity at the local and regional scales, resulting in frequent Clementsian and Gleasonian structures (Lopez‐Delgado et al., 2019).

### 4.2 Environmental and spatial effects on metacommunity

At the different spatial scales, both environment and spatial factors exerted varying influences on soil nematode metacommunity. At the local scale, environmental and spatial effects did not show significant effects on the metacommunity. However, environmental heterogeneity and dispersal limitation were important driving forces in shaping nematode metacommunity at the regional scale. Dispersal limitation became increasingly important at the larger spatial scale. This result is consistent with our second hypothesis. Dispersal limitation is generally considered to be important at the larger spatial scale, because significant geographic barriers (e.g., rivers, mountains) might restrict the dispersal of organisms, leading to segregation of genera into different metacommunities (Jocque et al., 2010). The ability and pattern of dispersal are also considered important variables that determine the coexistence and type of metacommunity (Cottenie, 2005). Nematodes often migrate to other sites through a passive dispersal pattern. However, this dispersal formed by passive transportation methods, such as wind, animal carriers and human activities, still has limitations at the larger spatial scale (Ptatscheck et al., 2018; Ptatscheck & Traunspurger, 2020). In addition, studies have shown that organisms with lower dispersal ability are more susceptible to the influence of spatial processes than those with higher dispersal ability (Rao et al., 2020). The spatial heterogeneity of nematode distributions from global to local scales is closely related to various environmental factors, dispersal processes and intrinsic community processes such as competition (Ettema & Wardle, 2002). The low dispersal ability of nematodes underscores the importance of dispersal limitation in the formation of metacommunities. This further confirms that dispersal limitations significantly affected nematode metacommunities at the regional scale, regardless of land cover types.

The Clementsian and Quasi‐Clementsian structures of nematode metacommunities at the regional scale also highlighted the important role of environmental heterogeneity in shaping the presence and absence of genera (Leibold & Mikkelson, 2002; Meynard et al., 2013). Although nematode metacommunities in all land covers were affected by environmental heterogeneity, the metacommunities were correlated with different environmental factors among different land cover types. The nematode metacommunities in the farmlands were more influenced by environmental factors than those in the grasslands and woodlands. This could be due to the impact of artificial management, which can lead to significant changes in soil organism communities, including alterations in biodiversity and limitations in energy transfer within the soil food web (Sanchez‐Moreno & Ferris, 2007; Yang et al., 2021; Zhang et al., 2017). As a result, the instability of the nematode metacommunity in farmlands increased, making it more susceptible to the effects of environmental factors. In this study, the nematode metacommunity in the farmlands was significantly correlated with soil moisture, pH, soil nitrogen and phosphorus. The findings confirm the importance of soil moisture and pH as key parameters controlling nematode activity in soil (Landesman et al., 2011; Shen et al., 2019). In addition, soil nitrogen and phosphorus are important indicators of soil fertility, and these soil nutrients directly or indirectly affect nematode metacommunity or even the soil food web by altering plant physiology or microbial activity (Cole et al., 2005; Zhang et al., 2022). Thus, soil properties are key environmental factors that affect nematode metacommunity in the studied agricultural ecosystems. For nematode metacommunity in the grasslands, soil nitrogen was the main driver. Nitrogen limitation is commonly observed in the grasslands of the Northeast China (Gao et al., 2019), and soil nitrogen is normally considered as a significant effect on the abundance of nematodes and their trophic groups in the grassland ecosystems (Xing et al., 2023). This suggests that soil nitrogen content could influence nematode community composition and ultimately affect the nematode metacommunity in the grasslands. The nematode metacommunity was significantly correlated with pH in the woodlands. Song et al. (2017) stated that at the global scale, soil acidity is a major factor that influences soil nematodes. Meanwhile, woodlands are characterized by abundant soil nutrients (Li et al., 2024). Therefore, it could be speculated that since there are sufficient nutrients in the woodlands, soil pH could be the main factor affecting nematode metacommunity in the woodlands.

### 4.3 Biological interactions effect on metacommunity

Consistent with our second hypothesis, biological interactions were found to be significant drivers of metacommunities at the local scale. Biological interactions have the potential to alter resource availability and the local abiotic environment, which can lead to contrasting effects on abundance, such as competition and facilitation (Lortie et al., 2004). In our study, we observed a similar pattern of variation between the degree of biological interactions and the abundance of plant parasites and bacterivores (Figure S1). In scenarios of higher abundance, individuals from different genera are more prone to compete for the same resources within limited space (Kraft & Ackerly, 2010), thereby intensifying biological interactions, particularly competition. Our findings indicate that biological interactions were primarily driven by dominant taxa across various land covers at the local scale.

Similar to the local scale, biological interactions were significant factors shaping metacommunities across the three land covers at the regional scale. While biological interactions were observed in both farmlands and natural land covers, our analysis revealed that the key interacting groups differed under each land cover type. In monoculture farmland sites, competition among plant parasites for limited resources might be intensified due to the absence of diverse vegetation (Decraemer et al., 2006). Our results supported this notion, indicating strong biological interactions among plant parasites in farmlands. In contrast, in the grasslands and woodlands, biological interactions were mainly expressed among bacterivores. It has been found that the abundance of bacterivores was greater in comparison with other trophic groups in the grasslands and woodlands (Figure S1). Bacterivores constitute a significant portion of the nematode population across various land cover types (da Silva et al., 2021; Mills & Adl, 2011). Microorganisms are the key energy source for nematode communities, and more of these resources were found in the grasslands and woodlands than in the farmlands (Biederman & Boutton, 2009). This might change the community composition and trophic structure of nematodes, thus leading to an increase in the abundance of bacterivores. With the high abundance of bacterivores, biological interactions were primarily characterized by competitive interactions among them. Moreover, our findings suggest that the robust biological interactions among nematodes facilitated their ability to track environmental heterogeneity.

## 5 CONCLUSION

The data obtained in this study indicate that the metacommunity structures of soil nematodes remained consistent across different land covers and spatial scales. At the local scale, biological interactions played a primary role in shaping soil nematode metacommunities, while at the regional scale, dispersal limitation and environmental heterogeneity also contributed, alongside biological interactions. These interactions varied among different trophic groups across land covers. Contrary to the initial hypothesis, nematode metacommunities exhibited significant coherence among land covers, likely due to their sensitivity to environmental gradients. Supporting the second hypothesis, biological interactions were indeed crucial at the local scale. However, at the regional scale, environmental factors and dispersal limitations were equally important. The competition for resources among nematodes drove these biological interactions, which were observed consistently across different land covers and spatial scales. Overall, understanding the role of biological interactions in soil nematode metacommunities is essential for deciphering their spatial patterns.

## AUTHOR CONTRIBUTIONS

**Ximei Niu:** Conceptualization (equal); data curation (equal); formal analysis (equal); investigation (equal); methodology (equal); validation (equal); writing – original draft (equal). **Ping Wang:** Investigation (equal); methodology (equal); supervision (equal); validation (equal); writing – review and editing (equal). **Zhijing Xie:** Investigation (equal); methodology (equal). **Meixiang Gao:** Investigation (equal). **Siru Qian:** Investigation (equal). **Ruslan Saifutdinov:** Writing – review and editing (equal). **Nonillon M. Aspe:** Writing – review and editing (equal). **Donghui Wu:** Investigation (equal); validation (equal). **Pingting Guan:** Funding acquisition (equal); investigation (equal); methodology (equal); project administration (equal); supervision (equal); writing – review and editing (equal).

## CONFLICT OF INTEREST STATEMENT

The authors have no conflict of interest to declare.

## Supporting information


Data S1.

Data S2.

## Data Availability

The data that support the findings of this study are available in the Data S1 and S2 of this article.
