# Supplementary material for: Soil nematode metacommunities in different land covers: Assessment at the local and regional scales
Source: Ecol Evol. 2024 May 23;14(5):e11468. doi: 10.1002/ece3.11468 (PMC11116945; doi:10.1002/ece3.11468)
Supplement: Supplementary file 1 — Data S1. [file ECE3-14-e11468-s002.docx]

**Supporting information**

**Table S1** Mixed effect model of land covers and sites and their interaction on environmental variables (means ± standard error).

| **Land covers** | **Sites** | **STC (g·kg^-1^)** | **STN (g·kg^-1^)** | **STP (g·kg^-1^)** | **pH** | **SM (g·kg^-1^)** | **ST (℃)** |
| --- | --- | --- | --- | --- | --- | --- | --- |
| Farmland | Heihe | 25.48±0.34a | 2.52±0.23a | 0.74±0.03a | 5.37±0.05b | 250.25±6.89a | 19.73±0.47c |
|  | Harbin | 15.08±1.01b | 1.53±0.09b | 0.41±0.01b | 6.60±0.32a | 196.12±10.79b | 26.69±0.26b |
|  | Lishu | 15.41±0.64b | 1.52±0.19b | 0.43±0.02b | 4.96±0.11c | 122.37±34.49c | 26.84±0.51b |
|  | Shenyang | 11.05±1.03c | 1.08±0.12b | 0.44±0.07b | 5.09±0.17bc | 150.98±7.08c | 29.09±0.35a |
| Grassland | Heihe | 33.68±3.04a | 3.39±0.16a | 0.62±0.05 | 5.40±0.07b | 261.35±8.02a | 22.66±0.05c |
|  | Harbin | 16.62±2.51ab | 1.50±0.19b | 0.37±0.01 | 7.23±0.17a | 130.60±15.09b | 23.58±0.64c |
|  | Lishu | 28.97±10.34ab | 2.74±1.02ab | 0.57±0.15 | 7.13±0.07a | 120.18±25.02b | 26.48±0.26b |
|  | Shenyang | 15.27±0.95b | 1.19±0.08b | 0.59±0.03 | 7.36±0.12a | 163.18±16.07b | 29.33±0.36a |
| Woodland | Heihe | 51.96±9.25a | 4.33±0.60a | 0.64±0.11a | 5.43±0.10b | 310.23±26.75a | 20.37±0.16c |
|  | Harbin | 27.03±1.87b | 2.30±0.19b | 0.41±0.02b | 7.02±0.05a | 141.57±10.82b | 23.92±0.44b |
|  | Lishu | 21.34±1.11b | 2.03±0.11b | 0.27±0.02b | 6.87±0.14a | 118.02±11.21b | 25.61±0.59a |
|  | Shenyang | 21.38±2.37b | 1.88±0.19b | 0.39±0.03b | 6.88±0.25a | 185.88±31.72b | 24.45±0.20b |
| Land covers | | **<0.001** | **<0.001** | **<0.001** | **<0.001** | **<0.001** | **<0.001** |
| Sites | | **<0.001** | **<0.01** | **<0.05** | **<0.001** | 0.26 | **<0.001** |
| Land covers x Sites | | 0.08 | 0.15 | 0.06 | **<0.001** | **<0.05** | **<0.001** |

Different lowercase letters indicate significant differences in environmental variables among sites under the same land cover; In bold are reported significant correlations.

**Table S2** Relationship between soil nematode metacommunity dissimilarity and environmental and spatial variables at the local scale based on the Mantel test.

| **Metacommunity** | | **STC** |  | **STN** |  | **STP** |  | **pH** |  | **SM** |  | **ST** |  | **Lon** |  | **Lat** |  |
| --- | --- | --- | --- | --- | --- | --- | --- | --- | --- | --- | --- | --- | --- | --- | --- | --- | --- |
|  |  | **r** | **P** | **r** | **P** | **r** | **P** | **r** | **P** | **r** | **P** | **r** | **P** | **r** | **P** | **r** | **P** |
| Farmland | Heihe | -0.33 | 0.91 | 0.55 | 0.06 | -0.38 | 0.96 | 0.13 | 0.32 | 0.74 | **<0.05** | -0.08 | 0.53 | 0.50 | 0.07 | 0.84 | 0.01 |
|  | Harbin | 0.53 | 0.07 | 0.24 | 0.17 | 0.10 | 0.34 | 0.60 | **<0.05** | 0.23 | 0.16 | 0.46 | **<0.05** | 0.63 | **<0.05** | 0.49 | 0.07 |
|  | Lishu | 0.58 | **<0.05** | 0.10 | 0.38 | 0.69 | **<0.01** | -0.02 | 0.56 | 0.77 | **<0.05** | 0.17 | 0.24 | 0.08 | 0.38 | 0.59 | 0.09 |
|  | Shenyang | 0.09 | 0.33 | 0.17 | 0.22 | -0.21 | 0.76 | 0.08 | 0.34 | -0.01 | 0.51 | -0.21 | 0.79 | 0.10 | 0.33 | -0.43 | 0.89 |
| Grassland | Heihe | 0.31 | 0.11 | -0.36 | 0.98 | 0.01 | 0.45 | 0.50 | **<0.05** | -0.07 | 0.57 | -0.15 | 0.71 | 0.51 | 0.09 | 0.48 | 0.11 |
|  | Harbin | -0.10 | 0.63 | 0.46 | 0.09 | 0.31 | 0.12 | 0.65 | 0.09 | -0.28 | 0.82 | 0.05 | 0.43 | 0.79 | **<0.05** | 0.70 | **<0.01** |
|  | Lishu | 0.73 | **<0.01** | 0.84 | **<0.01** | 0.45 | 0.09 | -0.27 | 0.88 | 0.77 | **<0.05** | -0.30 | 0.91 | 0.75 | **<0.05** | 0.90 | **<0.01** |
|  | Shenyang | 0.37 | 0.09 | 0.43 | 0.05 | 0.28 | 0.15 | -0.21 | 0.83 | 0.55 | **<0.05** | -0.06 | 0.52 | 0.03 | 0.47 | 0.73 | **<0.05** |
| Woodland | Heihe | 0.40 | 0.08 | 0.15 | 0.27 | -0.13 | 0.68 | 0.42 | 0.07 | -0.15 | 0.68 | -0.40 | 0.95 | 0.24 | 0.22 | 0.44 | 0.09 |
|  | Harbin | -0.19 | 0.80 | -0.37 | 0.98 | -0.33 | 0.97 | 0.08 | 0.37 | 0.40 | 0.10 | -0.21 | 0.84 | 0.54 | 0.08 | 0.66 | 0.05 |
|  | Lishu | 0.29 | 0.08 | -0.06 | 0.62 | 0.80 | 0.03 | 0.90 | **<0.01** | 0.54 | 0.05 | -0.10 | 0.62 | -0.23 | 0.86 | 0.84 | **<0.05** |
|  | Shenyang | -0.01 | 0.51 | 0.26 | 0.16 | 0.20 | 0.22 | 0.45 | 0.07 | 0.08 | 0.38 | 0.14 | 0.33 | -0.41 | 0.98 | -0.31 | 0.81 |

The Spearman correlation was used for this analysis; significant correlations are reported in bold. Abbreviations: STC - soil total carbon; STN - soil total nitrogen; STP - soil total phosphorus; SM - soil moisture; ST - soil temperature; Lon - Longitude; Lat - Latitude.





**Figure S1** Abundance of the trophic group of soil nematodes (ind.100g^-1^dry soil) at the local and regional scale. (A) local farmlands; (B) local grasslands; (C) local woodlands; (D) various land cover types at the regional scale. Abbreviations: NPP - the abundance of plant parasites; NFF - the abundance of fungivores; NBF - the abundance of bacterivores; NOP - the abundance of omnivores-predators. (A)(B)(C), Different lowercase letters indicate significant differences among the abundance of trophic group nematodes under the same site (P < 0.05); (D), Different lowercase letters indicate significant differences among the abundance of trophic group nematodes under the same land cover (P < 0.05).
